# Comparison of Physical Activity and Sedentary Behaviour between Schoolchildren with Cystic Fibrosis and Healthy Controls: A Gender Analysis

**DOI:** 10.3390/ijerph18105375

**Published:** 2021-05-18

**Authors:** Alexandra Valencia-Peris, Jorge Lizandra, Irene Moya-Mata, Fernando Gómez-Gonzalvo, Silvia Castillo-Corullón, Amparo Escribano

**Affiliations:** 1 Department of Teaching of Musical, Visual and Corporal Expression, University of Valencia, 46022 Valencia, Spain; jorge.lizandra@uv.es (J.L.); irene.moya@uv.es (I.M.-M.); 2 Department of Education, University CEU Cardenal Herrera, 12006 Castellón, Spain; fernando.gomez4@uchceu.es; 3 Pediatric Pulmonology Unit, Hospital Clínico Universitario de Valencia, 46010 Valencia, Spain; castillo_sil@gva.es (S.C.-C.); aescribano@separ.es (A.E.); 4 Department of Obstetrics and Gynecology, University of Valencia, 46010 Valencia, Spain

**Keywords:** exercise, sports, chronic disease, respiratory health, adolescence

## Abstract

The purpose of this study was to examine differences in sports participation and the levels of physical activity (PA) and sedentary behaviour (SB) between schoolchildren with cystic fibrosis (CF) and a healthy control group (CG) taking into account the gender variable. PA and SB were measured with an accelerometer for 7 consecutive days in 44 children (24 girls; 11.0 (3.2) years) with CF and 45 age-, sex-, and socioeconomic status-matched controls (24 girls; 11.1 (3.0) years). CF patients and CG did not differ in moderate-to-vigorous PA (54 (31) vs. 59 (27) min/day respectively) or in SB (558 (106) vs. 553 (92) min/day respectively). There were no differences in meeting the PA guidelines between both groups (CF: 36.4% vs. CG: 42.4%). Gender analysis revealed that boys were more active and met more PA guidelines than girls regardless of the group (CF or CG), girls with CF being the least active group (only 16.7% met PA guidelines). A possible compensatory effect was found between SB and PA only in the CF sample, as for each minute/day spent in SB the odds of meeting PA guidelines decreased by 34%. These findings suggest that promoting a reduction in SB is as important as promoting PA in the CF population, especially in girls. Health caregivers, coaches, teachers, or parents could offer appealing supervised and unsupervised physical activities, foster the adoption of active lifestyles, or incorporate PA into daily routines to improve the health of CF schoolchildren.

## 1. Introduction

The achievement of sufficient physical activity (PA) and rational sedentary behaviour (SB) for children and adolescents is a prevalent public health concern [1] and is especially relevant for young patients with cystic fibrosis (CF) [2,3]. CF is a progressive autosomal-recessive disease that predominantly occurs in individuals of European descent and affects over 72,000 people worldwide [4]. Due to a defect in the CF transmembrane conductance regulator gene, CF produces abnormally viscous secretions and results in obstruction of the airways, inflammation of the pancreas and organ failure that could end in premature death [5]. Fortunately, early diagnosis and improvements in therapy and knowledge of the illness have significantly increased the mean survival rates [6].

Although the severity of the disease has different effects on tolerance of exercise, an active lifestyle is clearly important [7,8,9]. Several studies have reported the beneficial effects of exercise training [3,8], especially as regards enhancing airway clearance and ion channel function transport [10], which can improve mucus hydration and clearance and slow down lung function decline [11,12]. Additional health benefits are the improvement of cardiorespiratory fitness by increasing the endurance and strength of respiratory muscles [9], bone mineral density [13], food intake and enhancing nutritional status [14]. These advances positively influence psychological and emotional dimensions such as self-confidence and sense of control [15], which stimulate the perception of a better quality of life [14]. Several reasons have thus made PA an important part of CF therapy and rehabilitation [3,9]. However, as unfortunately happens with healthy children and adolescents [16], the vast majority of young people with CF are not meeting the current guidelines for children and adolescents of performing at least 60 min of daily moderate to vigorous PA (MVPA) [8,17] as well as limiting the time spent in sedentary activities [18,19]. Inactivity also tends to increase during adolescence which, added to the long periods of sedentary time, is related not only to general metabolic, cardiovascular and psychological problems [20,21], but to the progress of both functional and physical impairments in the CF population [22].

The literature describes specific barriers for the participation of young people with CF [23]. A qualitative study by Denford et al. [15] reported tiredness and lack of time of both caregivers and adolescents as the main obstacles, and also the sense of vulnerability associated with the acceptance of the disease and dissatisfaction with PA. Other relevant barriers are the lack of understanding of the disease by teachers and healthcare professionals, which, together with parental reluctance to expose their children to strenuous exercise because of the fear of potential negative effects on their health [24], induce in children and adolescents a sort of disempowerment, loss of independence and self-imposed isolation that could be behind the inefficacy or lack of motivation in fitting PA into their daily routine [25,26].

Epidemiological studies have compared the prevalence of PA and SB in CF patients and healthy subjects, although the results caused a certain controversy, since some showed no significant differences in PA [19,27], others clearly found lower MVPA levels in CF patients than in their healthy counterparts [18,28], particularly if they had moderate or severe lung disease or impaired nutritional status [14,29], and others found differences depending on age and PA intensity [30]. There is little evidence in the literature on the effect of SB on PA for CF children and adolescents. Recent studies by Mackintosh et al. [19,31] found a competition for time during the week with low-intensity PA on weekdays to compensate for increased SB at the weekends. Another important gap in epidemiological studies on PA and SB in CF patients is that few of them incorporate gender as an important and independent variable in the comparison of (in)active behaviours. In fact, the few studies with gender comparison point to the lower participation of girls [14,27,32] in line with the healthy population [16]. Unfortunately, these studies had limited sample sizes and this precluded analysis of gender differences in habitual activity, so that there is a need to expand sample size and deepen these relations, especially in the CF context. The aim of this study was thus to examine differences in sports participation, SB and PA levels between CF children and a healthy control group (CG), including the gender effect. We hypothesized that CF children would be significantly more sedentary and less active than children from the CG, with differences by gender in both groups and between groups.

## 2. Materials and Methods 

### 2.1. Sample

A cross-sectional study of 44 patients with CF and 45 healthy peers aged 6–17 years was carried out throughout 2018. CF participants were recruited by screening the medical records of two CF Reference Units of two hospitals in the city of Valencia to select schoolchildren under treatment for CF. After the corresponding pneumologist provided consent, the subjects were enrolled in the study if they met all of the inclusion criteria: outpatient diagnosed using a genetic test for CF and treated at the aforementioned hospital, abnormal sweat chloride test (>60 mmol/L), boy/girl aged 6–17 years, and one or more of the following: exocrine pancreatic insufficiency, pulmonary disease symptoms or CF history in siblings or cousins. Exclusion criteria were: severe lung deterioration (FEV_1_% <30), unstable clinical condition (i.e., hospitalized, poor nutritional status) or any condition (e.g., muscle–skeletal disorder) impairing exercise testing. Forty-five sex-, age- and socioeconomic status-matched healthy students from primary and secondary schools of the same region as the hospitals were selected from a cohort of 118 participants as a CG. All the healthy participants stated that they had not suffered from nervous system and/or musculoskeletal disorders that could prevent their involvement in regular PA.

### 2.2. Instruments and Ethical Considerations

Bioelectrical impedance (BIA; model UM-076, Tanita, Tokyo, Japan) was used to determine body mass (kg) to the nearest 0.01 kg and percentage of fat, and a height stadiometer (model 1-S-B, 6615, company, Mediwaves Inc., Sonipat, Haryana, India) was used to measure height.

Socioeconomic status was measured by the Family Affluence Scale II [33], which reflects the family’s material resources and specific purchasing power. Participants completed this self-reported questionnaire with assistance from the research staff as necessary. Responses to each of the four items were added, achieving a number between 0 (lowest purchasing power) and 9 (highest purchasing power). 

Data regarding sports participation was obtained from a question asking for the physical activity/sports practiced. For some analyses, the participants were categorised according to their participation in the following variables: Organized PA (Yes/no), Swimming activities (Yes/No), Individual sports (Yes/No), Team Sports (Yes/No) and Individual and Team Sports (Yes/No). While individual sports refer to physical activities and sports which can be practised/played individually (i.e., skating, fighting sports, equestrianism, jumping, jogging), team sports were those in which participants were organized into opposing teams (i.e., football, basketball, handball, hockey). 

Participants were instructed to wear the Actigraph GT3X accelerometer (Actigraph LLC, Pensacola, FL, USA) on their right hip during all waking hours over 7 days. Accelerometers were only to be removed during bathing, water activities, and at bedtime. Raw accelerometer activity counts were processed in Actilife software version 6 (Actigraph LLC, Pensacola, FL, USA). Accelerometer data had a sampling frequency of 30 Hz and were collected as continuous count data that were divided into 15-second periods, with age-adjusted Freedson cut-points for determining moderate PA (2220–4135 counts per minute), vigorous PA (≥4136 counts per minute) and MVPA in youth [34]. As in previous studies [18,19], sedentary time was defined as <100 counts per minute, and the rest of the time was classified as light PA (100–2219 counts per minute). To report SB and PA outcomes accurately, expected motionless periods needed to be differentiated from periods of not wearing the accelerometer. Sustained periods of 20 min of consecutive zeros were used to define non-wearing time [35]. Participants who did not wear the accelerometer for at least 9 h in 3 days were not included in the study [36,37].

CF patients and their families were informed by their pneumologist about the nature of the study and provided with directions on the use of the accelerometer during a weeklong period in their routine hospital visits. Those who agreed to participate then started to wear the accelerometer and the research group visited the home of each participant one week later to remove the device and collect the rest of the measures. Measurements in the CG were performed in their own schools, where the researchers travelled to carry out the field work. 

Written informed consent and assent were obtained from parents/guardians and participants, respectively. The study was conducted in accordance with the Declaration of Helsinki and the protocol was approved by the Ethics Committee of the University of Valencia (H1488452044602) and authorized by the schools which participated.

### 2.3. Data Analysis

After the coding, cleaning, and grouping of the data, different statistical analyses were carried out using SPSS (version 26.0; IBMCorp, Armonk, NY, USA) with the α level set at *p* < 0.05. Descriptive statistics were obtained and expressed as frequencies and percentages, and mean and SD. Shapiro-Wilk test of normality was performed to check the assumption of a normal distribution, and because several variables did not meet this assumption, the values were transformed by calculating the square root. 

For continuous variables, between-group differences in anthropometric data were assessed using independent Student’s t-tests. Inter- and intra-group differences in sedentary time and different intensities of PA were assessed using analysis of variance (ANOVA) by group (CF/CG), gender and age group. For categorical variables (PA variables and PA guidelines accomplishment), Chi-square tests of independence were used with Cramer’s V to measure the size effect. The corrected standardized residuals were calculated to identify categories with significant differences (corrected standardized residuals ± 1.96). A binomial logistic regression was carried out to ascertain the likelihood of meeting PA guidelines according to sociodemographic and behavioural variables. The odds ratio was established with the confidence interval standing at 95%, and goodness of fit was tested with the Hosmer and Lemeshow test, using R^2^ estimates to gauge the percentage of explained variance.

## 3. Results

The main descriptive data for the study groups are provided in Table 1. The CG and the CF group did not differ in sociodemographic and anthropometrical characteristics, with the exception of height, which was significantly greater in the CG (*p* < 0.05).

Patients with CF and healthy controls did not differ in the type of PA and sports participation, as they usually engaged in organized PA and predominantly in individual sports. However, significant differences were observed in the number of activities and sports reported (*χ*^2^_(2)_ = 10.079; *p* < 0.05; Cramer’s *V* = 0.337). The study of the corrected standardized residuals revealed that more children with CF (22.7%) engaged regularly in three or more activities than youth from CG (4.4%) and were also less represented in the category of engaging in one activity than the children of the CG (29.5% CF children vs. 57.8% CG children). Significant differences were found also in the type of activity or sport practised (*χ*^2^_(2)_ = 6.741; *p* < 0.01; Cramer’s *V* = 0.275), where CF patients took part more in both individual and team sports (27.3%) than the CG children (6.7%). 

### 3.1. Patterns of Physical Activity and Sedentary Behaviour

PA and SB data are shown in Table 2. No significant difference was found between the CF patients and the CG in terms of average of wearing time per day (*M*_CF_= 863 min·day^−1^ vs. *M*_CG_ = 849 min·day^−1^, respectively). The results of the current study did not find significant differences between the diverse intensities of PA and SB between CF patients and the CG. Analyses based on gender variable were then conducted. The results of the univariate ANOVAs for PA and SB between the four groups (CF girls, CF boys, CG girls, CG boys) did not reveal significant differences in daily minutes of sedentary time and light PA. In both groups (CF and CG), moderate PA, vigorous PA and MVPA were significantly higher in boys than girls (intra group differences *p* < 0.001). In particular, boys devoted half an hour more than girls to daily MVPA in both groups. Significant inter-group differences were also detected in MPA, VPA and MVPA (*p* < 0.05), but never between participants of the same gender belonging to different groups. This means that CF boys were more active than the CG girls, and boys from the CG were also more active than girls in the CF group at all PA intensities. 

**Figure 1:**
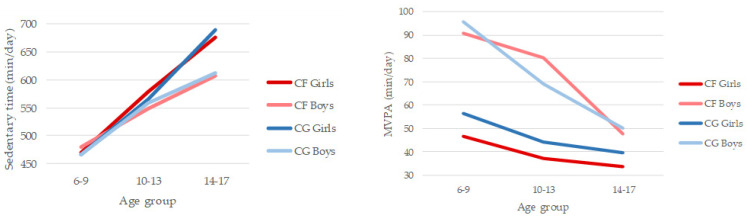
Patterns of daily sedentary behaviour and moderate-to-vigorous physical activity compared between cystic fibrosis (CF) and control group (CG) by gender and age group.

In the 6–17 age range sedentary time increased by 2 h and 40 min per day in children with CF and 3 h and 47 min in the CG. On the other hand, MVPA decreased by 13 min per day in children with CF and 47 min per day in children from CG. Results of the 2 (CF group/CG) × 2 (gender) × 3 (age group) ANOVA for SB indicated significant main effects for age group (*F*_(2,77)_ = 19.958, *p* < 0.001; *η*^2^ = 0.341) (Figure 1). Post hoc tests showed that all the age groups differed significantly, with SB becoming higher as children got older, especially in girls in both groups (CF and CG). No other significant differences were observed. Results of the 2 (CF group/CG) × 2 (gender) × 3 (age group) ANOVA for MVPA indicated significant main effects for gender (*F*_(2,77)_ = 22.592, *p* < 0.001; *η*^2^ = 0.227) and age group (*F*_(1,77)_ = 5.962, *p* < 0.01; *η*^2^ = 0.134) (Figure 1). Boys (*M*_boys_ = 73 min/day) were significantly more involved in PA, engaging in MPVA for 30 min more per day than girls (*M*_girls_ = 43 min·day^−1^). CF girls were the least active in the three age groups. Post hoc tests showed that younger children (6–9 years; *M* = 67 min·day^−1^) also differed significantly from older children (14–17; *M* = 40 min·day^−1^), the drop being more pronounced in boys. No other significant differences were observed. 

### 3.2. Accomplishment of PA Guidelines

**Figure 2:**
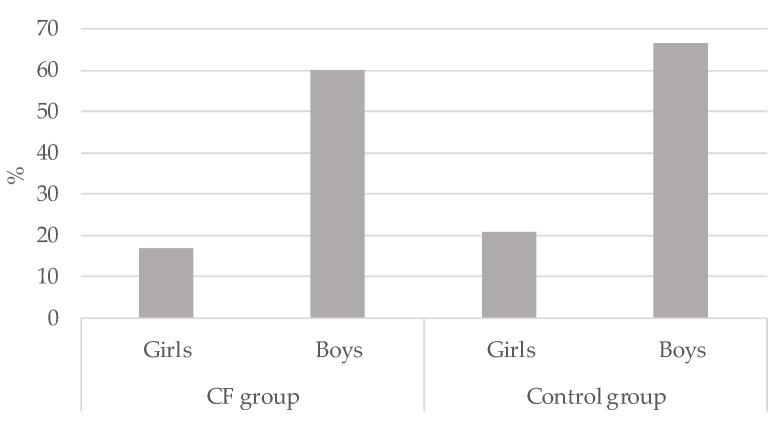
Percentage of physical activity guidelines accomplishment in cystic fibrosis (CF) children and control group by gender.

Overall, 36.4% (*n* = 16) vs. 42.2% (*n* = 19) of children in the CF and CG respectively met the MVPA guidelines (*p* > 0.05). Significant differences were observed when Chi-square tests were conducted to compare gender. As can be seen in Figure 2, boys met PA guidelines more than girls in the CF group (*χ*^2^_(2)_ = 8.85; *p* < 0.01; Cramer’s *V* = 0.449) as well as in the CG (*χ*^2^_(2)_ = 9.64; *p* < 0.01; Cramer’s *V* = 0.463). The group who less accomplished PA guidelines were the CF girls (16.7%) 

Finally, the studied variables were used to analyse the likelihood of meeting PA guidelines in each group (Table 3). For CF children, being a boy (eight times more likely than girls) and devoting less time to SB were significantly (*p* < 0.05) related to accomplishing PA guidelines, i.e., for each minute/day spent in sedentary activities, the odds of meeting PA guidelines decreased by 34%. However, in the CG, only being a boy was a predictor of being physically active (*p* < 0.05). Boys from the CG were 9.59 times more likely to meet the guidelines than healthy girls. 

## 4. Discussion

This study examined objective PA and sedentary patterns in children and adolescents with CF in comparison with a healthy CG focusing on a gender analysis. CF patients and children from CG did not differ in any level of PA nor in SB. Neither were there differences in meeting the PA guidelines between both groups. However, significant differences were observed between girls’ and boys’ PA levels and PA guidelines accomplishment, regardless of the group (CF or CG), where girls were less active than boys, and girls with CF were the least active group. 

Patients with CF and healthy controls did not differ in the type of PA and sports participation, as both engaged similarly in organized PA (72.7% vs. 80% respectively) and predominantly in individual sports (65.9% vs. 57.8% respectively). However, CF children reported participating more in both individual and team sports than children from the CG (27.3% vs. 6.7%). Besides, significant differences were shown in the quantity of physical and sports activities practiced by children with CF compared to CG. Of those with CF, 22.7% reported participating in three or more (un)structured physical activities or sports compared with 4.4% of the children in the GC. In this regard, as habitual PA is considered a part of standard CF care [8], CF children could participate more in a wider range of physical activities and sports than their counterparts. These differences in the number of sports or unstructured PA engagement may also be related to the influence of their peers on the one hand and their parents’ positive perception of PA and its benefits for health on the other, prioritizing a quantitative criterion over a qualitative criterion when encouraging an active lifestyle in their children. In a previous study [27], significant differences were found by gender in team sports participation, both in CF group (64.3% boys vs. 40.7% girls) and the CG (69.6% boys vs. 53.4% girls), with boys being more involved in sports activities than girls. 

In agreement with some previous studies [19,27,38] and contrary to others [18,30,39], no significant differences were observed in PA patterns and SB between CF children and the healthy CG. In a similar study conducted in Spain in 2014 [18], the authors highlighted lower levels of MVPA and less sedentary time in CF patients compared with healthy controls. Some studies detected only differences in vigorous activities, with CF children being less active than the healthy groups [30], but not in sedentary time [39]. However, as in this study, a recent systematic review and meta-analysis [40] concluded that CF children and adolescents have similar MVPA and sedentary time as controls. In fact, this study also did not find any differences between percentages of accomplishment between CF children (36.4%) and the CG (42.2%). The percentage of accomplishment in the CF group is very close to the 38.9% found in British children with CF [19], but far from the 2.1% reported in a Spanish sample [18], and from the 90% detected in another sample of British CF patients [41]. Although these studies used the same objective method of measuring PA levels (accelerometers), few studies have been conducted on this population, with small samples and high heterogeneity related to the different instruments used, to obtain properly robust information when comparing studies and samples [40,42]. In spite of the scarcity of studies, we must not lose sight of how CF young patients should engage in PA and sports as part of their therapy. According to Swisher et al. [8], children and adolescents with CF should: (a) enjoy a daily variety of activities for 60 min, preferably with family or friends; (b) between 30–60 min of the daily MVPA should be done at least at 70% of maximal heart rate, especially if practiced for airway clearance (must also cough to clear secretions); (c) take part in all activities that use body weight to strengthen muscles and bones (at least twice per week) for children and engage in formal resistance training 2–3 times per week per muscle group incorporating limb and trunk muscles for adolescents. However, it is important to highlight that counselling on PA choices which best fit children and adolescent’s interests and abilities will foster long-term adherence and adoption of a physically active lifestyle. It is also noteworthy that, in conjunction with CF patients, professionals set goals that allow them to experience positive experiences, success and the benefits of exercise, even if they are not ready for the recommended duration or intensity.

In both groups (CF and CG) there was a steep decline in MVPA and a noticeable increase in SB between children aged 6 and adolescents aged 17. These differences have also been observed in healthy adolescents [43], although there is recent evidence indicating that not all young people share the same trajectories [44]. In the CF sample, the study of Britto et al. [27] reported that for all categories of activity (vigorous activity, physical education lessons and sports participation), early and middle adolescents with CF were still much more likely to participate than were older adolescents. The authors reported a marked drop in vigorous PA (OR = 4.5) and sports team participation (OR = 6.5) from early to late adolescence. As higher levels of habitual PA in CF adolescents are associated with slower rate of decline in FEV_1_ [45], families and professionals need to continue to encourage the PA and sports practice which best fit their interests taking into account that for them, in addition to the changes and concerns typical of this period, puberty is often delayed and they may feel embarrassed by physically lagging behind their peers [8]. The unique concerns of these patients, such as coughing, expectorating, flatulence and body image concerns may make adolescents with CF even more likely to reduce their PA. Although adherence to all care guidelines can be challenging at this period, professionals need to consider ways for the adolescent to have a choice in the types of physical activity, timing and interests to maximize the chance of adherence to the guidelines. Regarding SB, no studies have been conducted with a CF sample that compare this behaviour during their childhood or adolescence.

This study also observed that SB can negatively affect the accomplishment of PA guidelines only in CF children. While there are studies conducted on healthy children that suggest that PA and SB may be asymmetrical, meaning that both behaviours may co-exist [46], in this study there could be some kind of competing effect in CF patients that does not affect the healthy controls. Although there is some inconsistency regarding the (in)dependence of these behaviours [47,48], one study conducted with CF patients exploring the “activitystat” hypothesis [39] demonstrated that, on any given day, every additional 10 min spent sedentary was associated with 2.4 min less sedentary time the following day. Although compensatory responses to PA and SB in CF patients are still unclear [31], this makes us think that it is just as important to reduce SB as it is to encourage PA in the CF population. In this regard, families and professionals could help young CF patients to develop strategies to overcome the barriers to being active (environmental, time management, medical treatments) and to include PA as a regular part of their daily lives. For example, they could include limiting screen time until after completing PA, do their homework riding a static bike, and incorporate bursts of activity into a school day, such as climbing stairs, active commuting or breaking up sedentary time. Encouraging patients to view websites and online chat groups targeted at teens with CF may also help them see the value of PA [8].

This study confirms that gender is a key factor in the differentiation of PA and SB [47,49], regardless of level of illness. Boys were more active than girls in moderate PA in all groups (19 min more per day in the CF group and 14 in the CG), vigorous PA (10 min more per day in CF and 16 in control) and MVPA (30 min more per day in CF group and 31 in CG). Inter-group differences were also found in all PA categories between participants of different gender, CF boys being more active than girls in the CG and CF girls less active than boys in the CG. Previous studies conducted with CF children also found boys reporting more regular PA than girls [32], either in the comparison group only [27] or in any group [38]. In the study of Selvadurai et al. [14], only pubescent CF boys were more active than pubescent girls, but no differences by gender were found in prepubescent children. Along the same lines, Puppo et al. [40] highlighted the fact that healthy girls tend to have less PA than healthy boys, as in CF children. According to these authors, one reason could be related to the fact that parents and teachers have lower expectations of girls engaging in regular PA than boys. Unfortunately, this expectation may influence girls’ intentions of becoming active and discourage them from adopting an active lifestyle. This is particularly detrimental for CF girls since participation in habitual PA has been shown to slow the rate of decline in lung function, and lower levels of habitual PA may partially explain the poorer survival rate of females with CF [32]. Although there are studies aimed at finding barriers to PA reported by CF youth [15], there are no special obstacles in the case of CF girls. However, there are qualitative studies which highlight gender differences in young CF adults about perceptions of living with CF. For example, Willis et al. [50] found that CF males were almost three times as likely to exercise than females and revealed that both males and females acknowledged an awareness of the importance of exercise for their health status. However, only men who did exercise declared any effects on competence, acceptance, autonomy and normality. As the aforementioned study concluded, it was more socially acceptable for CF males to be involved in PA and sports than females, despite it being associated with more frequent coughing and spitting due to increased lung activity. These results are also in line with the study conducted by Berge et al. [51], who found that over half of the CF female participants were less active than boys and found that they were concerned with body image. As Slater and Tiggemann reported [52], body image concerns may contribute to reducing adolescent girls’ rates of participation in sports and other physical activities. For these reasons, specific PA programmes should be addressed to girls in general and for CF girls in particular.

Although this study has numerous strengths, such as the objective measurement of PA and SB, its precise matching of healthy counterparts and its gender perspective, it is important to acknowledge certain limitations. First of all, its cross-sectional design precludes causal inferences on the associations among PA, SB and sociodemographic factors and limits the possibility of determining changes over time in all these variables. Secondly, the lack of data regarding involvement in swimming activities (in which 24.7% participated) could have underestimated the PA data registered in these participants. Thirdly, the CF patients’ clinical grade and lung function (FEV_1_) could be taken into account to provide more insights into their PA and SB trajectories from childhood to adolescence and for comparisons with the CG [8,14]. Finally, as according to the literature the type of day is another factor that affects PA participation [18,19], it would be of great interest to include this variable in future studies. 

## 5. Conclusions

In conclusion, the present study has demonstrated that children with CF behave in a similar way to a healthy CG in terms of daily levels of PA and SB, sports participation or in meeting PA guidelines. However, SB seems to negatively influence PA guidelines accomplishment only in CF children. The results also revealed gender as being a determining factor for engagement in PA and sports regardless of suffering CF. In both the CF and control groups, girls were less active and less likely to meet PA guidelines than boys, while CF girls emerged as the least active group of the sample. In view of these results, prescribed programmes which help to promote PA and reduce SB should be fostered in CF children, taking into account their gender and age. As CF girls are more vulnerable than boys in terms of PA participation, which could negatively affect their health status and quality of life, it is essential to carry out specific and enjoyable PA programs for them which minimize the existing social and psychological barriers to the practice of PA and at the same time maximize the health benefits of exercise. Thus, healthcare professionals, families, coaches and physical education teachers should be aware of CF conditions and promote an appealing offer of supervised and unsupervised physical activities, especially for CF girls. Adopting an active lifestyle would also be helpful in reducing the imbalance among PA and SB and in giving parents the decisive role of leading by example.

## Figures and Tables

**Table 1:** Characteristics of the sample.

	All (*N* = 89)	CF Group (*N* = 44)	Control Group (*N* = 45)	*p*
Sociodemographic variables				
Gender (girls), *n* (%)	48 (53.9)	24 (54.5)	24 (53.3)	0.909
Age (years), *M*(SD)	11.0 (3.1)	11.0 (3.2)	11.1 (3.0)	0.816
Socioeconomic status, *M*(SD)	5.4 (1.8)	5.3 (2.01)	5.5 (1.7)	0.503
Anthropometric variables, *M*(SD)				
Height, cm	146.1 (16.8)	**142.4 (16.3)**	**149.7 (16.7)**	**0.041**
Body mass, kg	40.1 (13.6)	37.5 (13.6)	42.6 (13.2)	0.063
BMI, kg.m^−2^	18.2 (2.9)	17.9 (3.1)	18.5 (2.8)	0.390
BMI, z-score	0.13 (1.1)	−0.03 (1.2)	0.28 (1.1)	0.211
Fat mass, %	22.0 (7.8)	22.9 (9.3)	21.3 (6.3)	0.433
PA variables, *n* (%)				
Organized PA	68 (76.4)	32 (72.7)	36 (80.0)	0.506
Number of activities/sports				
None	18 (20.2)	10 (22.7)	8 (17.8)	**0.018**
1	39 (43.8)	**13 (29.5)**	**26 (57.8)**	
2	20 (22.5)	11 (25.0)	9 (20.0)	
≥3	12 (13.5)	**10 (22.7)**	**2 (4.4)**	
Type of activity/sport				
Swimming	22 (24.7)	14 (31.8)	8 (17.8)	0.125
Individual sports	55 (61.8)	29 (65.9)	26 (57.8)	0.430
Team sports	29 (32.6)	15 (34.1)	14 (31.1)	0.764
Individual and team sports	15 (16.9)	**12 (27.3)**	**3 (6.7)**	**0.009**

CF: Cystic Fibrosis; M(SD): Mean (standard deviation); BMI: Body mass index; PA: Physical activity. Significant differences in bold.

**Table 2:** Differences in physical activity and sedentary behaviour between cystic fibrosis patients and control group by gender.

	CF Group			Control Group			*F*	*p*	*η* ^2^
	All	Girls	Boys	All	Girls	Boys			
Sedentary time (min·day^−1^)	558 (106)	574 (96)	538 (117)	553 (92)	566 (99)	539 (83)	0.794	0.501	0.027
Light PA (min·day^−1^)	240 (64)	224 (61)	260 (64)	230 (63)	220 (61)	241 (64)	1.710	0.171	0.057
Moderate PA (min·day^−1^)	39 (20)	30 (13) ^a,c^	49 (22) ^a,b^	41 (15)	35 (10) ^a,b^	49 (16) ^a,c^	8.968	**<0.001**	0.240
Vigorous PA (min·day^−1^)	15 (13)	10 (7) ^a,c^	20 (17) ^a,b^	17 (15)	10 (5) ^a,b^	26 (17) ^a,c^	7.855	**<0.001**	0.217
MVPA (min·day^−1^)	54 (31)	40 (19) ^a,c^	70 (36) ^a,b^	59 (27)	45 (14) ^a,b^	76 (29) ^a,c^	10.566	**<0.001**	0.272

Data expressed as Mean (SD). CF: Cystic fibrosis; PA: Physical activity; MVPA: Moderate-to-vigorous physical activity. Significant differences in bold. ^a^ Significant differences intra-group.^b,c^ Significant differences inter-group.

**Table 3:** Binomial logistic regression to predict the accomplishment of physical activity guidelines by group.

	CF Group		Control Group	
	OR	95% CI	OR	95% CI
Gender (boys)	8.02	(1.63–39.42)	9.59	(1.99–46.28)
Age	0.94	(0.70–1.24)	0.88	(0.61–1.26)
Sedentary time (min/day)	0.66	(0.44–0.99)	0.65	(0.34–1.22)

CF: Cystic fibrosis. CF model: *R*^2^ = 0.03, model *χ*_2_^2^ = 16.856, *p* < 0.01. Control Group model: *R*^2^ = 0.02, model *χ*_2_^2^ = 17.462; *p* < 0.01. Significant effects are shown in bold (*p* < 0.05).

## Data Availability

The data presented in this study are available on request from the corresponding author. The data are not publicly available due to ethical reasons.
